# Sex Differences in the Clinical Features and Outcomes of Patients with Acute Coronary Syndrome Treated with Two Generations (Absorb and Magmaris) of Bioresorbable Vascular Scaffolds

**DOI:** 10.3390/jcm10173768

**Published:** 2021-08-24

**Authors:** Adrian Włodarczak, Piotr Rola, Marek Szudrowicz, Magdalena Łanocha, Mateusz Barycki, Jan Jakub Kulczycki, Alicja Gosiewska, Karol Turkiewicz, Maciej Lesiak, Adrian Doroszko

**Affiliations:** 1 Department of Cardiology, The Copper Health Centre (MCZ), 59-300 Lubin, Poland; wlodarczak.adrian@gmail.com (A.W.); marek.szudrowicz@gmail.com (M.S.); jan.jakub.kulczycki@gmail.com (J.J.K.); kturkiewicz@mcz.pl (K.T.); 2 Department of Cardiology, Provincial Specialized Hospital in Legnica, Iwaszkiewicza Str. 5, 59-220 Legnica, Poland; mateusz.barycki@gmail.com; 3 Adalbert’s Hospital, 61-144 Poznan, Poland; magdalena.lanocha@ump.edu.pl; 4 Faculty of Mathematics and Information Science, Warsaw University of Technology, 00-662 Warsaw, Poland; alicjagosiewska@gmail.com; 5 1st Department of Cardiology, Poznan University of Medical Sciences, 61-491 Poznan, Poland; maciej.lesiak@skpp.edu.pl; 6 Department of Internal Medicine, Hypertension and Clinical Oncology, Wroclaw Medical University, 50-556 Wroclaw, Poland; adrian.doroszko@gmail.com

**Keywords:** sex differences, acute coronary syndrome (ACS), bioresorbable scaffold (BRS), Magmaris, Absorb, gender-related prognosis, percutaneous coronary intervention (PCI), coronary artery disease (CAD)

## Abstract

Background: Despite the developments in percutaneous coronary interventions (PCI), women are still more likely than men to have unfavorable outcomes after PCI performed in Acute Coronary Syndrome (ACS). The mechanisms of this phenomena are not fully understood. Potential benefits of bioresorbable scaffolds (BRS) may be particularly expressed in the female population. Nevertheless, the data available currently are inconsistent and limited. This study evaluated the gender-related differences in the short-term clinical outcomes in ACS patients treated with implantation of two generations of BRS (first generation, Absorb; second generation, Magmaris). Methods: The study was divided into two arms. To the first one, we qualified 160 patients with ACS treated with PCI who received 210 Absorb scaffolds. The second arm was composed of 193 patients with ACS who underwent PCI with Magmaris implantation. Results: There were no significant sex-related differences in primary endpoints (cardiovascular-death, myocardial infarction, in-stent thrombosis) or principal secondary endpoints (of target-lesion failure, scaffold restenosis, death from any reason, other cardiovascular events) in either generation of BRS in a 1-year follow-up. Conclusions: Both genders tended to have a similar outcome in routine clinical practice following BRS implantation due to ACS. The magnesium bioresorbable scaffold (Magmaris) early outcome seemed to be more favorable in comparison to the Absorb scaffold.

## 1. Introduction

Cardiovascular disease (CVD) and, particularly, coronary artery disease (CAD) continue to be the leading cause of mortality and morbidity, as reported worldwide annually [1]. Acute manifestation of the CAD, acute coronary syndrome (ACS), remains among the most common causes of percutaneous coronary interventions (PCI) in everyday clinical practice [2]. From the earlier days of balloon angioplasty and bare-metal stents, women have been at a higher risk of poor PCI clinical outcomes in comparison to men [3]. Despite the undeniable development in the therapy of ACS, which was observed following the introduction of the second DES generation, women are still more likely than men to have an unfavorable outcome [4].

The exact mechanisms that stand behind these differences remain unclear. Several factors are postulated to have an impact on these phenomena. Female patients are generally older [5], are characterized by longer delay from onset of ACS symptoms until the first medical contact, and are more likely to have multiple comorbidities accompanied with less aggressive pharmacotherapies and decreased rate of invasive procedures [6]. Additionally, the pathophysiology of ACS seems to be different: Women have a higher incidence of nonobstructive CAD and plaque erosion in contrary to the frequently observed plaque rupture in the male population [7]. The pathophysiology of plaque erosion is multifactorial and strongly related to the endothelial dysfunction which activates pathogenic cascades, including immunological processes, such as transient sympathovagal imbalance resulting in increased inclination to coronary vasospasm. Since metallic scaffold has an unfavorable impact on the arterial healing process, by inducing local inflammatory response [8], bioresorbable vascular scaffolds were designed as a device to enable overcoming these limitations [9]. This technology was assumed to bring an equality to metallic scaffold vessel support during the acute phase of healing, and disappears after its useful function in preventing recoil and constrictive remodeling, which allows restoring physiological vessel functionality.

These potential benefits of BRS devices may be particularly expressed in the female population; however, data regarding this subject are inconsistent and limited. A study that compared prognosis in male and female patients implanted with a bioresorbable scaffold was mainly focused on Absorb (Abbott Vascular, Santa Clara, CA, USA) scaffolds. Shreenivas et al. [10], in a pooled meta-analysis of four studies, suggested similar outcomes in the female and male groups. However, Baquet et al. [11] and Kerkmeijer et al. [12] suggested that clinical outcomes tended to be better in females compared to males. To date, there are no data regarding the second generation of BRS, a magnesium scaffold called Magmaris (Biotronik, Berlin, Germany). Therefore, we designed this study to evaluate the difference between genders in the short-term clinical outcome (1 year) in ACS patients treated with implantation of two generations of BRS (Magmaris vs. Absorb).

## 2. Materials and Methods

### 2.1. Study Population

**Figure 1:**
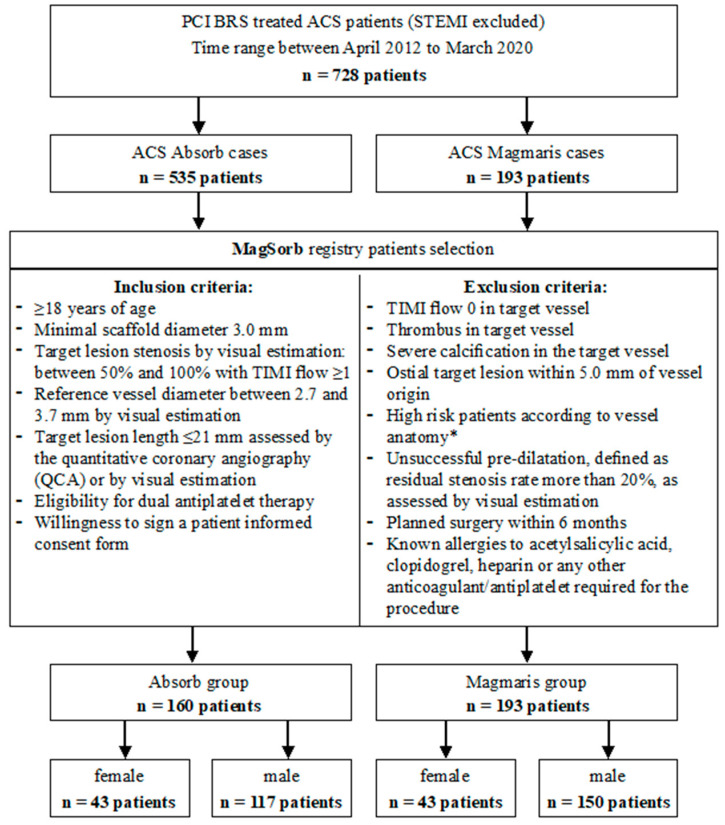
Study inclusion and exclusion criteria. PCI, percutaneous coronary intervention; BRS, bioresorbable scaffolds; ACS, acute coronary syndrome; STEMI, ST-segment elevation myocardial infarct; CABG, coronary artery bypass graft. * Left main disease or equivalent, three-vessel disease, or multi-vessel disease disqualification from CABG by local Heart Team.

To the study (MagSorb Registry), we included subjects undergoing PCI at the Department of Cardiology of Cooper Health Center in Lubin between April 2012 and March 2020 with subsequent implantation of two generations of BRS (Magmaris and Absorb) due to symptoms of ACS, with the exclusion of patients with ST-segment elevation myocardial infarct (STEMI). The indication for percutaneous coronary intervention (PCI) was based on clinical symptoms and the presence of significant angiographic CAD. The decision for selecting the BRS was left up to the operators. Magmaris scaffolds were implanted in concordance with the current recommendations and consensus of experts [13] as well as inclusion and exclusion criteria dedicated to our ACS-Magmaris Registry (Figure 1). Patients selected to the Absorb group were retrospectively chosen out of all ACS Absorb’s cases implanted in our Cardiology Department (*n* = 535 patients). All patient included in the study had to meet the inclusion and exclusion criteria mentioned in Figure 1. Additionally, the scaffolds had parallel in size to Magmaris group (diameter 3.0 mm or 3.5 mm). All pooled data were analyzed by sex and device modality. In this two-arm (Absorb and Magmaris) study we evaluated the sex-specific safety and efficacy for each device separately.

All the data obtained by standardized questionnaire were collected by the trained medical staff and entered retrospectively into an electronic database.

### 2.2. Device and Procedures

Implantation of the Absorb (Abbott Vascular, Santa Clara, CA, USA) everolimus-eluting poly-l-lactic BRS and Magmaris (Biotronik, Berlin, Germany) sirolimus-eluting magnesium bioabsorbable coating (BIOlute) Poly-l-Lactide (PLLA) BRS was carried out according to the above mentioned recommendations [13] after mandatory, successful (without any significant (over 20% of diameter) residual stenosis in angiographic assessment) lesion preparation with a balloon catheter (balloon:artery ratio 1:1 size by angiographic assessments with coexisting vessel diameter in a range from 2.7 mm up to 3.7 mm). Post-dilatation with an NC balloon, sized at least equal or up to 0.5 mm longer than the size of the scaffold, was obligatory. Post-dilatation was performed at high pressure (not less than 16 atm) If necessary, intravascular imaging was performed due to the decision of the operators. Standard pharmacotherapy was carried out following the current ESC/ESH guidelines for non-ST-segment elevation myocardial infarction (NSTEMI) [14,15]: double antiplatelet therapy for 12 months.

### 2.3. Endpoints and Definitions

The primary outcome was composed of death from cardiac causes, myocardial infarction, and stent thrombosis. The principal secondary outcome was a target-lesion failure (TLF) defined as cardiac death, target vessel myocardial infarct (TV-MI), or target lesion revascularization (TLR). Other secondary outcomes included scaffold restenosis, death from any reason, cerebrovascular episodes, and all kinds of revascularization procedures as well as myocardial infarction. Myocardial infarction was defined according to the Fourth Universal Definition of Myocardial Infarction [16].

### 2.4. Statistical Analysis

The analyses were conducted using the R language [17]. Continuous variables were characterized with their mean and standard deviation, while frequencies were used for categorical variables. The patients were compared between groups with the nonparametric, two-sample Mann–Whitney’s Test for continuous variables and Fisher’s Exact Test for categorical variables. Bonferroni correction was applied to adjust for multiple comparisons. The *p*-values ≤ 0.05 were accepted as a threshold for statistical significance.

## 3. Results

### 3.1. Patient Characteristics

To the first arm we qualified 160 patients with acute coronary syndrome (ACS) treated with percutaneous intervention who received *n* = 210 Absorb scaffold. The female Absorb group consisted of 43 patients who had undergone interventions in 46 lesions, with subsequent *n* = 55 Absorb scaffold implantation. This group was compared with 117 males with 123 hemodynamically significant lesions, into which *n* = 155 Absorb BRS scaffold were implanted. The second arm of the study was composed of 193 patients with acute coronary syndrome who underwent PCI with implantation of Magmaris BRS (*n* = 204). In this group, we enrolled 150 males and 43 females. Table 1 summarizes the baseline clinical characteristics of both groups.

In this study women with ACS were older than men in both groups (Magmaris, 66.86 vs. 63.16, respectively, *p* = 0.108; Absorb, 69.12 vs. 64.68, respectively; *p* = 0.060). However, the differences were not statistically significant. In the female Absorb group, unstable angina was more frequently observed than NSTEMI (55.8% vs. 33.3%, respectively, *p* = 0.011) (44.1% vs. 66.6%, respectively, *p* = 0.011). In the Magmaris group, women had a lower prevalence of past MI (16.2% vs. 34.6%, respectively, *p* = 0.024) and history of previous percutaneous intervention (23.2% vs. 45.3%, respectively, *p* = 0.013) as well as initial left ventricle systolic function (EF-LV 63.91% ± 12.12 vs. 59.21% ± 10.34, respectively, *p* = 0.007), and serum creatine levels (73.78 ± 19.27 vs. 87.03 ± 22.10, respectively, *p* < 0.001). Furthermore, in this BRS-Magmaris group, women had higher than men LDL-C (2.92 ± 1.23 vs. 2.38 ± 1.11, respectively, *p* = 0.048) and total cholesterol (5.10 ± 1.31 vs. 2.38 ± 1.11, respectively, *p* = 0.024) levels on admission. Neither of these dependences was observed in the group treated with Absorb scaffold implantation.

### 3.2. Index Procedure Variables

The characteristics of the PCI procedures performed in both tested generations of BRS were heterogeneous. The only observed, statistically significant difference in the Magmaris group was a tendency to more frequent postdilation with a balloon catheter equal to the size of a previously implanted stent (34.8% vs. 10.6%, respectively, *p* = 0.001). In the Absorb group, males more often received 3.5-mm scaffolds (83.7% vs. 67.5%, respectively, *p* = 0.001) and received greater amounts of contrast (174.3 ± 59.3 vs. 154.8 ± 52.4, respectively, *p* = 0.047)**.** In the Magmaris group, men received more contrast and radiation; however, these differences were just beyond the statistical significance (*p* = 0.062 and *p* = 0.067). Perforations were more frequent in the Absorb arm, although no gender-related relationship was observed. All procedural-related data are presented in Table 2.

### 3.3. Clinical Outcomes

All the data related to clinical outcomes are pooled in Table 3. There were no statistically significant differences in sex- and device-related clinical outcomes. However, in the Absorb group, in a 1-year follow-up, we observed a higher rate of the primary outcome in the female population (11.63% vs. 6.84%, respectively, *p* = 0.338). Similar relationships were observed in the short-term follow-up (30 days). Females with Absorb were more likely to have target lesion failure (9.3% vs. 4.27%, respectively, *p* = 0.252) mainly due to an increased number of target vessel MI (9.3% vs. 4.27%, respectively, *p* = 0.252) and target lesion revascularization (6.98% vs. 3.42%, respectively, *p* = 0.387). No analog interactions were found in the Magmaris group. As can be seen in Table 4, there were no statistically significant differences between the female Magmaris and Absorb groups. However, rates of the primary outcome and principal secondary outcome were higher in the female Absorb group compared to Magmaris, both in the short-term follow-up of 30 days (Primary outcome, 4.65% vs. 0%, respectively, *p* = 0.494; and Principal Secondary outcome, 4.65% vs. 0%, respectively, *p* = 0.494) as well as the longer observation period of a 1-year follow-up (Primary outcome, 11.63% vs. 2.3%, respectively, *p* = 0.202; and Principal Secondary outcome, 9.3% vs. 0%, respectively, *p* = 0.116).

## 4. Discussion

To the best of our knowledge, this is one of the first “real-world” studies investigating the impact of gender on the outcome of ACS-PCI performed using the ABSORB bioresorbable stents in comparison to the second generation of BRS Magmaris, a novel magnesium scaffold.

Women with ACS in comparison to men had been at a higher risk for poor clinical outcomes following PCI, particularly in the short-term follow-up period after primary PCI [18,19]. However, the exact mechanisms of these sex-specific differences remain unclear and are probably multifactorial [20].

One of the main pathophysiological differences found in autopsy studies is a higher female proportion in plaque erosion cases compared to ruptures [21]. Erosion is also more common in non-ST segment elevation than in ST-segment elevation myocardial infarction [22]. Recently, a potentially new conservative (non-scaffold-related) treatment paradigm appeared [23]. However, the temporary nature of bioresorbable scaffolds may create an option for a new pathophysiology-dependent treatment model for ACS.

Although coronary artery disease in particularly acute coronary syndrome remains an important cause of mortality in females, women are underrepresented in clinical trials focused on this subject. Additionally, in our study women were a minority (86 vs. 267). However, thanks to the proper design of the study it was possible to reduce to a minimum the potential confounders that may have had an impact on results and obtained mainly scaffold-sex-related outcomes.

One of the major risk factors that affects prognosis is age. It has been proven that, in general, women who undergo ACS-PCI are older than men [24] and this observation is consistent with the data collected in our study. However, in our study population these differences were statistically insignificant.

Moreover, increased prevalence of traditional risk factors (hypertension, DM t.2, hyperlipidemia) of ACS has been described as one of the factors that affect outcomes of PCI in the female population [25]. However, in the population recruited to our study, this phenomenon did not occur and for some risk factors was even reversed (females in the Magmaris group had a significantly lower prevalence of history of MI and previous percutaneous intervention compared to males).

Another important risk factor that is postulated as a female’s predictor of an unfavorable outcome is the lower size of the vessels in the female population [26]. By selecting patients with a lesion that was in the large coronary artery (minimum scaffold diameter: 3.0 mm) we managed to avoid this disturbing factor. Moreover, thanks to the exclusion of patients with cuprite lesions in smaller vessels, we managed to avoid an independent predictor of adverse outcomes after Absorb scaffold implantation [24,27]. At the same time, we obtained a heterogeneous research group: The second generation of BRS is available only in the 3.0- to 3.5-mm size.

To perform aggressive predilation and postdilation for appropriate stent optimization we managed to reduce a described [20,24,25] inequality in the intensiveness of treatments between sexes. Additionally, such an approach became an essential part of the BRS implantation due to experts’ recommendation [13]. Moreover, guideline-recommended pharmacological therapies for CAD tended to be administered less often in women than in men [20,24,25]. We vanquished this disturbing factor in our study on principal pharmacotherapy after ACS-PCI (dual antiplatelet therapy, statin, and ACEI/ARB) that was conducted due to actual recommendations, and there were no statistically significant differences between men and women.

In our strictly device-oriented study, we analyzed gender-related differences in the short-term outcomes in both BRS-generation cohorts. We found no statistically significant differences in Primary and Principal Secondary outcomes. This finding is consistent with the observation Shreenivas S. et al. [10] made for Absorb scaffolds. However, there was a noticeable trend of poorer outcomes (primary outcome and principal secondary outcome) in the female cohort. This was particularly notable in the Absorb group. These data are in opposition to the finding of Baquet et al. [11], who reported statistically insignificant higher rates of target lesion failure, target lesion restenosis, and stent thrombosis in males with implanted Absorb BRS. Similar unfavorable outcomes in males were part of the Absorb-related study of Kerkmeijer et al. [12], who showed sex-related higher rates of TLF, TV-MI, and TLR. However, in both mentioned studies, women had lower complexity of CAD (significantly lower SYNTAX score and shorter lesions) with other coexisting, mentioned sex-related dissimilarities, which could affect the obtained results.

There are currently no data on the sex-related differences regarding the second generation of the BRS magnesium scaffold. In our study, implantation of Magmaris in ACS in routine clinical practice showed favorable outcomes in both genders and did not disclose any differences among males and females. Nevertheless, these studies demonstrated a borderline interaction in the female population between the two generations of BRS, suggesting an increased risk of device-oriented events in the Absorb female group. Similar observations were made when we compared Absorb to “classical” DES [28], which might suggest that Magmaris might overcome previously observed imperfections of BRS scaffold technology. Improvement in radial strength, scaffold resorption, and deliverability may establish BRS in routine clinical practice, especially in the female population in whom outcomes are less encouraging. However, future long-term, big numbers-based observations focused particularly on comparisons to the second generation of BRS and DES are necessary

### Limitations

This study has several limitations. Data were collected retrospectively in the short observation period (1-year follow-up). Additionally, in a relatively small group, females were underrepresented, which could exert to some extent an effect on the statistical power. Moreover, this study is related only to BRS scaffolds without comparison to classical DES. 

## 5. Conclusions

Both genders, when receiving equal in-hospital and post-discharge management, seem to have similar short-term outcomes in routine clinical practice after BRS implantation during ACS. Data obtained in our study might suggest that implantation of the second generation of bioresorbable (Magmaris) in comparison to the first-generation (Absorb) scaffolds is associated with more favorable outcomes. However, there is a strong need for large-number, multicenter, prospective studies to elucidate fully the sex-related differences in terms of the short- and long-term outcome of PCI in ACS.

## Figures and Tables

**Table 1:** Baseline clinical characteristic of both study arms.

	Magmaris Group			Absorb Group		
	Male *N* = 150	Female *N* = 43	*p*-Value	Male *N* = 117	Female *N* = 43	*p*-Value
Age [years]	63.16 ± 9.42	66.86 ± 6.41	*p* = 0.108	64.68 ± 9.24	69.12 ± 10.29	*p* = 0.060
Unstable angina	23	7	*p* = 0.816	39	24	*p* = 0.011
NSTEMI	127	36	*p* = 0.816	78	19	*p* = 0.011
Diabetes mellitus type 2	58	14	*p* = 0.592	43	18	*p* = 0.585
Oral anti-diabetic treatment	50	8	*p* = 0.089	31	16	*p* = 0.240
Insulin	8	6	*p* = 0.088	12	2	*p* = 0.355
Hypertension	131	40	*p* = 0.418	95	36	*p* = 0.819
Hyperlipidemia	117	35	*p* = 0.833	98	35	*p* = 0.812
Atrial Fibrillation	6	3	*p* = 0.420	8	3	*p* > 0.999
Post PCI status	68	10	*p* = 0.013	44	14	*p* = 0.584
Primary Diagnosis of MI	52	7	*p* = 0.024	40	10	*p* = 0.248
LVEF	59.21 ± 10.34	63.91 ± 12.12	*p* = 0.007	56.42 ± 13.36	53.33 ± 12.79	*p* = 0.150
Total Cholesterol (mmol/L)	4.46 ± 1.30	5.10 ± 1.31	*p* = 0.024	5.08 ± 1.33	5.07 ± 1.35	*p* = 1
LDL (mmol/L)	2.38 ± 1.11	2.92 ± 1.23	*p* = 0.048	2.92 ± 1.14	2.87 ± 1.15	*p* = 1
Triglycerides (mmol/L)	1.87 ± 1.98	1.67 ± 0.85	*p* = 1	2.94 ± 6.40	1.78 ± 1.03	*p* = 0.265
Creatine (µmol/L)	87.03 ± 22.10	73.78 ± 19.27	*p* < 0.001	88.51 ± 17.07	85.66 ± 17.67	*p* = 0.391

Abbreviations: NSTEMI, no ST-Elevation Myocardial Infarction; PCI, percutaneous coronary intervention; MI, Myocardial Infarction; EF-LV, Ejection fraction of the left ventricle.

**Table 2:** Procedural characteristics of both study arms.

	Magmaris Group			Absorb Group		
Procedural Characteristic	Male *N* = 150	Female *N* = 43	*p*-Value	Male *N* = 117	Female *N* = 43	*p*-Value
Treated vessel: LAD	64	16	*p* > 0.999	64	24	*p* > 0.999
LCX	37	12	*p* > 0.999	16	8	*p* > 0.999
RCA	46	15	*p* > 0.999	43	14	*p* > 0.999
IM	3	0	*p* > 0.999	0	0	-
Predilatation balloon						
- Mean diameter (mm)	3.22 ± 0.28	3.27 ± 0.25	*p* = 0.290	2.98 ± 0.36	2.99 ± 0.20	*p* = 0.777
- Mean pressure (atm)	17.69 ± 0.80	17.66 ± 0.76	*p* = 0.745	1.11 ± 4.13	0.28 ± 1.83	*p* = 0.259
Average scaffold number	1.05 ± 0.23	1.07 ± 0.26	*p* = 0.686	1.32 ± 0.47	1.28 ± 0.45	*p* = 0.583
Scaffold diameter						
- 3.0 (mmm)	71	17	*p* = 0.390	58	26	*p* = 0.284
- 3.5 (mm)	87	29	*p* = 0.293	98	29	*p* = 0.029
Average scaffold length (mm)	20.83 ± 3.14	20.70 ± 3.71	*p* = 0.913	22.77 ± 4.84	22.60 ± 4.78	*p* = 0.864
Postdilatation balloon						
- Mean diameter (mm)	3.54 ± 0.31	3.52 ± 0.25	*p* = 0.762	3.49 ± 0.30	3.45 ± 0.24	*p* = 0.507
- Mean pressure (atm)	17.80 ± 0.6	17.75 ± 0.67	*p* = 0.522	18.00 ± 2.61	18.93 ± 2.20	*p* = 0.074
- 0.0 mm greater than scaffold	16	15	*p* = 0.001	52	18	*p* = 0.858
- 0.25 mm greater than scaffold	106	24	*p* = 0.096	44	20	*p* = 0.364
- 0.5 mm greater than scaffold	28	4	*p* = 0.169	21	5	*p* = 0.469
Contrast Volume (mL)	154.8 ± 65.0	139.4 ± 66.5	*p* = 0.061	174.3 ± 59.3	154.8 ± 52.4	*p* = 0.047
Dose of radiation (mGy)	1098 ± 711	911 ± 637	*p* = 0.067	1629 ± 882	1339 ± 738	*p* = 0.062
OCT guided PCI	32	9	*p* > 0.999	10	4	*p* > 0.999
Number of edge dissections	4	3	*p* = 0.186	7	2	*p* > 0.999
- treated with BVS	2	1	*p* = 0.533	5	1	*p* > 0.999
- treated with DES	2	2	*p* = 0.215	2	1	*p* > 0.999
Perforation of vessel treated	0 (0%)	0	-	2	1	*p* > 0.999
- with covert stent	0 (0%)	0	-	1	1	*p* = 0.467
- with prolonged balloon inflation	0 (0%)	0	-	1	0	*p* > 0.999
Side branch occlusion	2	0	*p* > 0.999	1	1	*p* = 0.467
Drugs: ASA	150	43	-	117	43	-
Clopidogrel	58	18	*p* = 0.726	87	35	*p* > 0.999
Ticagrelor	92	25	*p* = 0.726	28	7	*p* > 0.999
Prasugrel	0	0	-	2	1	*p* > 0.999
Statin	147	43	*p* > 0.999	117	42	-
ACEI/ARB	119	42	*p* > 0.999	11	39	*p* > 0.999

Abbreviations: OCT, optical coherence tomography; PCI, percutaneous coronary intervention; ASA, acetylsalicylic acid; ACEI, Angiotensin-converting enzyme inhibitors; ARB, Angiotensin receptor blockers.

**Table 3:** Clinical outcomes in both study arms.

	Magmaris Group			Absorb Group		
Clinical Outcomes	Male *N* = 150	Female *N* = 43	*p*-Value	Male *N* = 117	Female *N*= 43	*p*-Value
30-Day Follow up						
Primary outcome: cardiac death, myocardial infarction, stent thrombosis	0 (0%)	0	-	3 (2.56%)	2 (4.65%)	*p* = 0.611
Principal secondary outcome: Target lesion failure (cardiac death, target vessel myocardial infract, target lesion-revascularization)	0 (0%)	0	-	3 (2.56%)	2 (4.65%)	*p* = 0.611
Death						
- Cardiac	0 (0%)	0	-	0 (0%)	0 (0%)	-
- Any	0 (0%)	0	-	0 (0%)	0 (0%)	-
Myocardial infarction						
- Target vessel	0 (0%)	0	-	3 (2.56%)	2 (4.65%)	*p* = 0.611
- Any	0 (0%)	0	-	0 (0%)	0 (0%)	-
Scaffold						
- thrombosis	0 (0%)	0	-	3 (3.7%)		*p* = 0.611
- restenosis	0 (0%)	0	-	0	0	-
Stroke	0 (0%	0	-	0 (0%)	0 (0%)	-
TIA	0 (0%)	0	-	0 (0%)	0 (0%)	-
Revascularization						
- Target lesion	0 (0%)	0	-	3 (2.56%)	2 (4.65%)	*p* = 0.611
- Target vessel	0 (0%)	0	-	3 (2.56%)	2 (4.65%)	*p* = 0.611
- Any	0 (0%)	0	-	3 (2.56%)	0	*p* = 0.564
1 Year Follow up						
Primary outcome: cardiac death, myocardial infarction, stent thrombosis	2 (1.33%)	1 (2.3%)	*p* = 0.533	8 (6.84%)	5 (11.63%)	*p* = 0.338
Principal secondary outcome: Target lesion failure (cardiac death, target vessel myocardial infract, target lesion-revascularization)	3 (2%)	0	*p* > 0.999	5 (4.27%)	4 (9.3%)	*p* = 0.252
Death						
- Cardiac	0(0%	0 (0%)	-	1 (0.85%)	0 (0%)	*p* > 0.999
- Any	1 (0.67%)	1 (2.33%)	*p* = 0.397	1 (0.85%)	1 (2.32%)	*p* = 0.467
Myocardial infarction						
- Target vessel	2 (1.33%)	0	*p* > 0.999	5 (4.27%)	4 (9.3%)	*p* = 0.252
- Any	2 (1.33%)	1 (2.33%)	*p* = 0.533	3 (2.56%)	1 (2.33%)	*p* > 0.999
Scaffold						
- thrombosis	0 (0%)	0	-	4 (3.42%)	2 (4.65%)	*p* = 0.661
- restenosis	2 (1.3%)	0	*p* > 0.999	1(0.85%)	1 (2.33%)	*p* = 0.467
Stroke	1 (0.67%)	1 (2.33%)	*p* = 0.397	3 (2.56%)	1 (2.33%)	*p* > 0.999
TIA	0	1 (2.33%)	*p* = 0.223	0 (0%)	0 (0%)	-
Revascularization						
- Target lesion	2 (1.33%)	0	*p* > 0.999	4 (3.42%)	3 (6.98%)	*p* = 0.382
- Target vessel	3 (2%)	0	*p* > 0.999	4 (3.42%)	4 (9.3%)	*p* = 0.212
- Any	14 (9.33%)	4 (9.30%)	*p* > 0.999	13 (11.11%)	3 (6.98%)	*p* = 0.561

Abbreviations: TIA, transient ischemic attack; PCI, percutaneous coronary intervention; ASA, acetylsalicylic acid; MI, Myocardial Infarction.

**Table 4:** Differences in clinical outcomes between Absorb and Magmaris female groups.

Clinical Outcomes	Magmaris Female *N* = 43	Absorb Female *N* = 43	*p*-Value
30-Day Follow up			
Primary outcome: cardiac death, myocardial infarction, stent thrombosis	0	2 (4.65%)	*p* = 0.494
Principal secondary outcome: Target lesion failure (cardiac death, target vessel myocardial infract, target lesion-revascularization)	0	2 (4.65%)	*p* = 0.494
Death			
- Cardiac	0	0 (0%)	-
- Any	0	0 (0%)	-
Myocardial infarction			
- Target vessel	0	2 (4.65%)	*p* = 0.494
- Any	0	0 (0%)	-
Scaffold			
- thrombosis	0	2	*p* = 0.494
- restenosis	0	0	-
Strok	0	0 (0%)	-
TIA	0	0 (0%)	-
Revascularization			
- Target lesion	0	2 (4.65%)	*p* = 0.49
- Target vessel	0	2 (4.65%)	*p* = 0.494
- Any	0	0	*-*
1 Year Follow up			
Primary outcome: cardiac death, myocardial infarction, stent thrombosis	1 (2.3%)	5 (11.63%)	*p* = 0.202
Principal secondary outcome: Target lesion failure (cardiac death, target vessel myocardial infract, target lesion-revascularization)	0	4 (9.3%)	*p* = 0.116
Death			
- Cardiac	0 (0)	0 (0%)	-
- Any	1 (2.33%)	1 (2.33%)	*p* > 0.999
Myocardial infarction			
- Target vessel	0	4 (9.3%)	*p* = 0.116
- Any	1 (2.33%)	1 (2.33%)	*p* > 0.999
Scaffold			
- thrombosis	0	2 (4.65%)	*p* = 0.494
- restenosis	0	1 (2.33%)	*p* > 0.999
Stroke	1 (2.33%)	1 (2.33%)	*p* > 0.999
TIA	1 (2.33%)	0 (0%)	*p* > 0.999
Revascularization			
- Target lesion	0	3 (6.98%)	*p* = 0.241
- Target vessel	0	4 (9.30%)	*p* = 0.116
- Any	4 (9.30%)	3 (6.98%)	*p* > 0.999

Abbreviations: TIA, transient ischemic attack; PCI, percutaneous coronary intervention; ASA, acetylsalicylic acid; MI, Myocardial Infarction.

## Data Availability

Data not included in manuscript available on request from corresponding author due to local law and privacy restrictions.
